# Detection of Viral Proteins in Human Cells Lines by Xeno-Proteomics: Elimination of the Last Valid Excuse for Not Testing Every Cellular Proteome Dataset for Viral Proteins

**DOI:** 10.1371/journal.pone.0091433

**Published:** 2014-03-11

**Authors:** Alexey L. Chernobrovkin, Roman A. Zubarev

**Affiliations:** 1 Department of Medical Biochemistry and Biophysics, Karolinska Institutet, Stockholm, Sweden; 2 SciLifeLab, Stockholm, Sweden; UGent/VIB, Belgium

## Abstract

Cell cultures used routinely in proteomic experiments may contain proteins from other species because of infection, transfection or just contamination. Since infection or contamination may affect the results of a biological experiment, it is important to test the samples for the presence of “alien” proteins. Usually cells are tested only for the most common infections, and most of the existing tests are targeting specific contaminations. Here we describe a three-step procedure for reliable untargeted detection of viral proteins using proteomics data, and recommend this or similar procedure to be applied to every proteomics dataset submitted for publication.

## Introduction

Cell lines are widely used as model systems in biology, especially in cancer research [1]. The number of available cell lines grows rapidly. Whereas the pioneering panel introduced by National Cancer Institute in 1990 contained 60 cancer cell lines (NCI-60), large-scale studies reported nowadays are dealing with panels as large as 639 [2] and 947 [3] cell lines.

One of the main motivations for working with cell lines is their supposedly identical behavior in different labs, provided the experimental conditions are similar or identical. This presumed reliable behavior is critical for reproducibility of scientific experiments, which is the backbone of the scientific method. During the recent survey it has been found that a substantial part of cancer papers were irreproducible [4]. One of the known issues leading to irreproducible results is the cell line contamination. Ten years ago, the German Collection of Microorganisms and Cell Cultures (DSMZ) found that 18% of 252 submitted cell lines were cross-contaminated with more than half of the contamination sources located within only six laboratories. Subsequent testing performed by DSMZ with extended number of cell lines showed that of 598 leukemia–lymphoma cell lines, 187 (31%) were contaminated with mycoplasma and/or a second cell line, with 38 (6%) of cell lines contaminated with both [5].

Cross-contamination remains a serious issue today [6]–[8]. The database of cross-contaminated or misidentified cell lines is freely accessible on the web (see go.nature.com/soppaj) and lists more than 400 cell lines. Many of the listed cell lines become contaminated at the source, so that all subsequent experimental work has become questionable [9]. This is why all cell banks now employ methods to confirm the identity and purity of the cell lines they distribute [10], whereas scientific journals and funding bodies make authentication testing compulsory [11]. While commonly used cell identification based on short tandem repeats does not provide complete authentication of a human cell line, novel methods based on SNP profiling [8] or cell proteomic footprints [12] are much more reliable.

In contrast to contamination by microbes and mycoplasma, which can be relatively easily detected, viral contamination presents a serious threat because of the difficulty to detect some viruses and the lack of effective methods of curing infected cell cultures [13]. Cell banks commonly test cell lines for several widespread viral infections by using PCR or RT-PCR. However, even certified cell lines can still be contaminated by viruses that do not lead to a cytopathic effect. At DSMZ, only tests detecting human pathogen viruses have been performed for human cell lines [13].

Viral infections can cause very significant consequences for biological research. In 2006, the telltale genetic signature of the virus XMRV has been detected in tissue samples taken from men with prostate cancer [14]. The initial work reporting a link between prostate cancer and XMRV infection has become highly cited, but most (although not all) subsequent studies have failed to confirm this association. Since the initial discovery, XMRV and MLV-related viral sequences resembling polytrophic MLVs (P-MLVs) have also been found in patients with chronic fatigue syndrome (CFS) [15]. The original paper published in 2006 has been retracted in December 2011 [14]. Recent re-evaluation of the original cohort/samples as well as the new ones showed that the association of XMRV with prostate cancer arose from laboratory contamination of clinical samples by an XMRV-infected LNCaP cell line [16].

This alarming example suggests that the problem of cell line contamination is widespread and it cannot be dismissed as a malpractice within a few poorly managed laboratories. The vast majority of current microbial or viral infection tests are based on PCR, ELISA and other methods targeting specific organisms. In contrast, untargeted omics techniques (genomics, transcriptomics and proteomics) provide complete or very significant coverage of the respective domain, practically eliminating the risk of missing a contaminant *in the dataset* provided the genome of the contaminant is known. However, the data analysis methods used today in e.g. shotgun proteomics of cell lines, are often blind to other organisms than the target one. The standard protocol simply discards the data that do not fit into genome of the host organism, thus ignoring, and in some cases misattributing, even significant contamination [17], [18]. In view of the above scary examples of misattribution, such a practice should be discouraged, and a better procedure needs to be found for interpretation of shotgun proteomics data.

In shotgun proteomics, proteolytic peptides are identified by comparing experimental tandem mass spectra of peptides with theoretical ones derived from the sequence databases [19]. Matching theoretical peptide to experimental spectrum has a probabilistic nature. Removing non-relevant sequences from the sequence database reduces the probability of random match between experimental and theoretical spectra, thus increasing the sensitivity of the method [20]. Thus, if there is no contamination, there is no reason to consider other species in the database search than the target one. However, choosing only one specific organism for matching the shotgun proteomics data amounts to assigning zero probability to the presence of other organisms in the sample. As has been demonstrated above, such an assumption can be dangerous. On the other hand, inclusion of several organisms in the database search amounts to assigning equal *a priori* probabilities to all proteins of all organisms, which reduces the search sensitivity and may lead to erroneous matches. Thus the simplest way to account for the possibility of viral presence, adding the sequences of all known viral proteins to the protein sequences database, is a faulty approach.

An opposite extreme is to ignore the host sequences in the proteomics data and focus on the viral ones, thus assigning zero *a priori* probability to host proteins and 100% - to viral proteins. Such an approach was utilized by Bromenshenk et al. [21], which resulted in their identifying peptides of Iridovirus and Nosema origin in North American honey bees. However, subsequent studies by Foster [22] and Knudsen and Chalkley [23] have proved findings of Bromenshenk et al. wrong, with the misidentification of viral proteins being caused by inappropriate usage of viral-only sequence database for mass-spectrometry data interpretation. In human proteomics, false positive identification of viral peptides is even more likely in the above approach given that up to 8% of human genome has viral origin [24], [25], and thus some human proteins exhibit high degree of homology to retroviral proteins.

In view of the deficiencies of the above extreme approaches, a balanced procedure is needed that assigns higher (but less than 100%) *a priori* probability to human sequences compared to viral sequences.

Here we describe such a balanced three-step procedure for reliable detection and relative quantification of viral proteins in proteomics data using a combination of standard approaches based on well-tested statistical models. Because of its simplicity and availability of proteomics analysis, the procedure can be easily adopted by most, if not all, cell labs. It can also be easily extended to non-viral contaminations. Moreover, proteome profiling can also confirm the identity of the cell line, which is another point of recent serious concerns [26].

The procedure consists of label-free proteomics analysis with subsequent identification of human as well as non-human proteins by the tandem mass spectra (MS/MS) of their peptides. We addressed the fact of unequal a priori probabilities for viral and human peptide matches by identifying all human-related MS/MS spectra first, leaving for subsequent identification only non-human data. The final stage of the procedure comprises quantitative estimation of viral protein abundances in comparison to the host proteome, to determine the severity of contamination.

The procedure can also be applied to already acquired data. A recent editorial in Nature Methods urges researchers to reprocess existing proteomics raw data “with new questions in mind…” [27]. Here we followed this advice and used published proteomics data. First, proof of principle is obtained by discovering expected viruses in cell lines known to be producing viral proteins (both cell line established by viral transfection and cell line known to be contaminated by virus). As a control, we analyzed proteomes of 58 NCI-obtained cell lines from the NCI-60 panel. This panel has previously been screened for envelope viral proteins and gene sequences related to xenotropic murine leukemia viruses (X-MLVs) [28] and only one cell line, EKVX, has been found infected by xenotropic murine leukemia virus. Finally, we applied the three-step procedure to other cell line proteomes analyzed by a reputable group [18] and discovered unexpected viral contamination in at least one of the reported 11 lines.

## Materials and Methods

### Data Sets

Mass-spectrometric data (raw data files) were provided: deep proteomics of eleven common cell lines [18] by Mann’s group (data were deposited at ProteomeCommons.org Tranche); sixty proteome datasets for NCI-60 cancer cell lines - by Kuster’s group [29] (data can now be downloaded from the web http://wzw.tum.de/proteomics/nci60). Both datasets comprise mass-spectra obtained from the whole cell lysates. Proteins were extracted from cells, digested with trypsin. Peptide mixtures where separated using 1D (reverse-phase liquid chromatography) or 2D protocol (strong cation exchange chromatography followed by reverse-phase liquid chromatography). Mass-spectra were acquired in data-dependent mode with CID or HCD fragmentation.

MS^2^ spectra were extracted and stored in Mascot generic file (mgf) format using in-house developed Raw2MGF software (available for non-commercial use upon request), with peak picking performed using Thermo Xcalibur centroiding. No filtering or preprocessing of raw MS/MS data was performed. For each cell line, a single mgf file containing all available MS/MS spectra was produced manually by concatenation of individual LC/MS data files.

### Peptides and Proteins Identification

Mascot 2.4.1 (Matrix Science, UK) MS/MS search engine was used. For the initial identification of human-related proteins and peptides, search was performed in the UniProt complete database of human protein sequences (release of January 2013; 87,638 records) combined with 248 common contaminant sequences (http://maxquant.org/contaminants.zip). At the second stage of identification, SwissProt viral protein database was used (January 2013; 16,317 records). Viral subset of SwissProt database contains manually curated sequences of proteins for **398** reference strains covering 370 viral genera [30], which makes it representative but non-redundant protein sequence database.

Trypsin specificity was used in MS/MS search with up to two missed cleavages. N-terminal acetylation and methionine oxidation were selected as variable modifications, while carbamidomethylation of cysteine was selected as a fixed modification. Precursor ion mass accuracy was set to 15 ppm, fragment ion mass tolerance to 30 mDa. Automatic decoy database search and built-in Percolator algorithm (version 2.01) [31] were used to rescore search results and calculate posterior error probabilities for each peptide-spectrum match. All results were filtered to achieve global FDR ≤1%.

### Quantification

To determine the normalized protein abundances, ion current based label-free quantification [32] was used with a minimum of two unique peptides. All raw files were reprocessed with MaxQuant version 1.3.7.4 with Andromeda as a database search engine. MaxQuant analysis included an initial search with a precursor mass tolerance of 20 ppm, the results of which were used for mass recalibration. In the main Andromeda search, precursor mass and fragment mass had an initial mass tolerance of 6 ppm and 20 ppm, respectively. The search included as variable modifications methionine oxidation and N-terminal acetylation, and carbamidomethyl cysteine as a fixed modification. Minimal peptide length was set to seven amino acids and a maximum of two missed cleavages was allowed. The false discovery rate (FDR) was set to 0.01 for peptide and protein identifications. In the case of several proteins identified with all common peptides, these were combined and reported as one protein group. To verify MaxQuant data, a more precise Quanti program was used designed in house [33], which yielded similar results.

## Results

**Figure 1:**
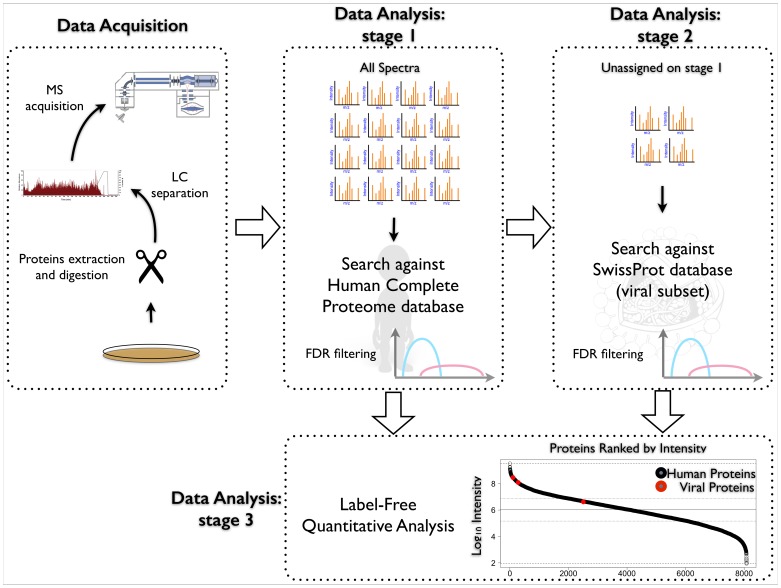
The suggested three-step workflow of shotgun proteomics analysis that includes contamination/infection detection. (1) conventional matching the experimental MS/MS spectra against the protein database of the host organism (human); (2) Unmatched MS/MS spectra and insignificant matches in terms of FDR are matched against viral protein database. (3) The presence of significant hits at the second step points to the potential infection/contamination of the sample. Label-free quantitative analysis of identified proteins (both host and contaminant) provides an estimation of the contamination level and thus severity of the problem.

The three-step procedure (Figure 1) utilizes in two first steps of MS/MS database searching, together with a standard determination of false positive detection rate (FDR), of which one search is conventional and another is a xeno-proteomics search for a foreign organism. On the third stage, label-free quantification is performed. First, the available proteomics MS/MS dataset is searched against the database of human protein sequences. All MS/MS spectra that fit to human sequences with a given FDR are then flagged. The remaining unassigned MS/MS spectra are then submitted to a second search, against a database of all known viral (or other foreign) proteins sequences. The presence of significant (in the FDR sense) matches indicates the presence of viral infection or contamination of the sample. The detected viral proteins are then quantified by one of the available label-free methods, and the proteins are ranked by their abundances. The rank of non-human proteins indicates the level of infection and the seriousness of its consequences for the cell.

### Proof-of-principle

HEK293 cell line has been established by transformation of normal human embryonic kidney cells with sheared DNA of adenovirus 5 and it is known to express viral proteins E1A and E1B [34]. The HEK293 proteome, as well as the other 10 proteomes in the cited study [18], was represented by 620,000±20,000 MS/MS spectra obtained from the authors. The first-stage search on HEK293 proteome by Mascot (MatrixScience, UK) attributed 361,736 of the 624,107 MS/MS spectra to human-originated tryptic peptides. The search results were rescored by Percolator and filtered at 1% FDR, yielding 7,970 human protein groups. The remained spectra were searched against the viral subset of the SwissProt database using a Mascot feature “re-search non-significant spectra”. The results were processed by Percolator as above, yielding three proteins (E1BS, E1BL and E1A) of human adenovirus 5 (Table S1). Using intensity-based label-free quantification, we estimated the relative abundance of viral proteins compared to the human cellular proteome (Table S2). E1BS and E1BL proteins were found to be among the top 100 most abundant proteins expressed by HEK293 cells. Thus, viral proteins can’t be ignored when doing even most “shallow” proteomics work. With this example, we established that our procedure successfully detects viral proteins that should be present in the cell “by design”.

To test whether the procedure can detect viral *contamination* that is almost surely present in the sampled cells, we analyzed proteomic data of EKVX cell lines from the NCI60 panel. The presence of viral infection in this cell line has been detected during the screening of the NCI60 panel for envelope proteins and gene sequences related to xenotropic murine leukemia viruses (X-MLVs) [28]. The shotgun proteomic analysis of the EKVX cell line described elsewhere [29] provided 144,926 MS/MS spectra, 62,450 of which were assigned by Mascot to the 3,613 human proteins. Using the proposed three-step approach, we identified both env (ENV_XMRV3) and pol (POL_XMRV3) viral proteins, which is consistent with the PCR-based results obtained on the same cell line in a different [28] study.

### Negative Control

Both envelope proteins and gag and pol DNA sequences observed by PCR in EKVX cell line, have not been detected in any other NCI60 cell line [28]. We used the proteomics data on the remaining 58 NCI-60 cell datasets [29] as a negative control for our procedure. The data quality (resolution in MS and MS/MS) for nine cell lines MCF7, M14, COLO205, CCRFCEM, U251, H460, PC3, SKOV3 and RXF393) was similar to that in Geiger et al. [18], whereas 49 cell lines were represented by low-resolution CID MS/MS spectra. We were able to observe in high-resolution MS/MS data from 6000 to 8000 human protein groups and 2800–4000 groups in low resolution MS/MS data. Not a single viral peptide was detected with ≤1% FDR in either of the 58 cell lines.

### Test Analysis

**Figure 2:**
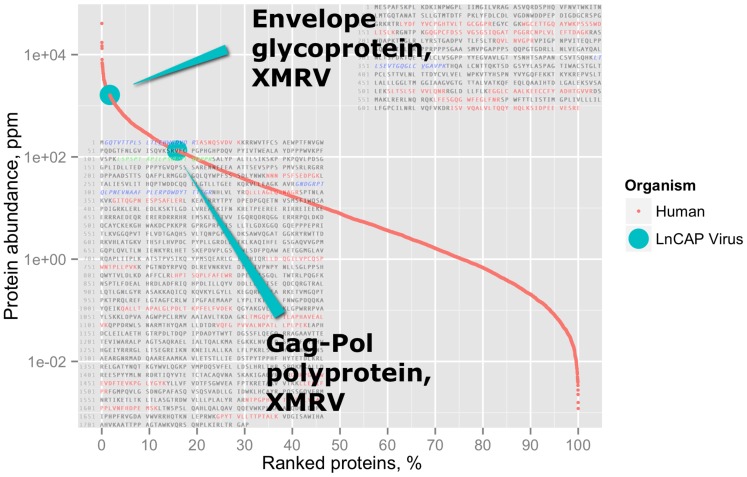
Expression levels of the XMRV viral proteins within the proteome of the human cell line LNCaP. Small red dots correspond to the host cell proteins, whereas the two identified viral proteins are marked as cyan circles. Detected sequences of tryptic peptides of the two viral proteins are marked with color: red - peptides mapped perfectly on the XMRV sequence; blue and green - mutated sequences.

The proteomes of the remaining 10 cell lines studied by Geiger et al. were interrogated. In the LNCaP cell line, we found two viral proteins belonging to the XMRV virus. The envelope glycoprotein (ENV_XMRV4) and Gag-Pol polyprotein (POL_XMRV4) compose full proteome of that virus (Table S3). The observed peptides represented a relatively low coverage of proteins sequences (13% and 25%, respectively), but were distributed along the whole protein sequence (Figure 2), indicating likely expression of fully functional viral polypeptides.

We also observed in the same dataset several peptides, which mapped to the proteins of other viruses homologues to the XMRV (Table S3). This may indicate that the virus presented in given LNCaP cell line differs from the one previously studied, which is expected due to the high mutation rate in retroviruses [35].

Label-free quantitative estimate by both MaxQuant and Quanti [33] showed that both POL_XMRV6 and ENV_XMRV6 are highly expressed in the LNCaP cell (Figure 2, Table S4). Thus the production of infectious viral particles could be expected and precautions must be taken while working with these contaminated cells.

MaxQuant does not currently allow for two-stage database search procedure; therefore, MaxQuant quantification was based on the independent search of MS/MS spectra against a concatenated database containing both human and viral proteins. As expected, such a database search yielded many false positive viral identifications due to the overestimation of the a priori probabilities for viral peptides. In total, proteins corresponding to 75 viruses including different bacteriophages, murine coronavirus, tobacco mosaic virus, etc., have been “identified” by MaxQuant (Tables S5, S6, Figure S1). Such a gross overestimation of the viral proteins presence by searching in a concatenated database validates our two-stage database searching approach as most relevant.

## Conclusions

In the 2012 paper that refuted the link between XMRV and prostate cancer, the clinical cancer samples have been shown to be contaminated with the XMRV-infected LNCaP cell line [16]. Using gag RT-PCR, the authors found XMRV in 2003 LNCaP cells from the Cleveland Clinic laboratory and no XMRV in the 2012 LNCaP cells from the UCSF Cell Culture Facility. Here we found that XMRV-infected LNCaP cells were still used in 2011–2012 on a different continent (upon the discovery, the laboratory in question, that did not report the origin of their cells in the cited work, was immediately notified). The widely used German cell line bank DSMZ, according to their web-site information, did not test their LNCaP cells for the XMRV infection.

Summarizing, every lab should take seriously the risk of cell contamination. The widely adopted in academia practice of sharing cell lines between colleagues (especially popular among young faculty members who are often stretched for funding) facilitates dissemination of contaminated cultures inside the institutes and beyond [9]. To trace cell dissemination and thus control their contamination, it would be helpful to report the cell origin in every published work.

Proteomics easily detects contamination with, in principle, any virus or other foreign organism.

Viral contamination differs from microbial contamination in terms of the proteome size: viral proteome can be represented by only a few proteins, while microbial proteome is much larger (>500 proteins). Thus, detecting viral contamination is more challenging. Direct transfer of convenient data-analysis approaches widely used in single-organism proteomics may result in “false alarm” events [22], [23]. It should also be taken into account that some human proteins exhibit high degree of homology to retroviral proteins. In such case standard target-decoy strategy may fail [36], so the special attention should be paid to the peptide-spectrum matches attributed to retroviral proteins. For instance, additional filtering based on delta score (score difference between the best and next best matches) could help reducing the amount of false positives. We also recommend manual rechecking of the MS/MS spectra and peptide assignments, as well as the uniqueness of the viral peptide sequences.

Given that our procedure is easy and uses standard proteomics software tools, there is no valid excuse left for not using the described method of detection (or similar) when proteomics data are available.

## Supporting Information

Figure S1 Histograms and density plots of Scores, Delta Scores and normalized Delta Scores (Delta Score divided by Score) for viral peptides identified in proteomes of eleven cell lines by Andromeda search engine at 1% FDR threshold by matching MS/MS spectra against concatenated human-viral sequence database. Abbreviations: HAdvC – human adenovirus C identified in HEK293 cell line, XMRV – xenotropic murine leukemia virus-related virus identified in LNCaP cell line, Other – other peptide-spectral matches from eleven cell lines uniquely attributed to viral peptides.
(PDF)

Table S1 Adenovirus 5 proteins and their peptides identified in HEK293 cell line by shotgun proteomics.
(XLSX)

Table S2 Human and viral proteins quantified in HEK293 cell line using intensity based label-free quantification.
(XLSX)

Table S3 XMRV proteins and their peptides identified in LNCaP cell line by shotgun proteomics.
(XLSX)

Table S4 Human and viral proteins quantified in LNCaP cell line using ion signal based label-free quantification.
(XLSX)

Table S5 Viral peptides identified in proteomes of eleven cell lines by Andromeda search engine at 1% FDR threshold by matching MS/MS spectra against concatenated human-viral sequence database.
(XLSX)

Table S6 Viral proteins identified in proteomes of eleven cell lines by Andromeda search engine at 1% FDR threshold by matching MS/MS spectra against concatenated human-viral sequence database.
(XLSX)
