# Supplementary figures and images for: Detection of Viral Proteins in Human Cells Lines by Xeno-Proteomics: Elimination of the Last Valid Excuse for Not Testing Every Cellular Proteome Dataset for Viral Proteins
Source: PLoS One. 2014 Mar 11;9(3):e91433. doi: 10.1371/journal.pone.0091433 (PMC3950186; doi:10.1371/journal.pone.0091433)

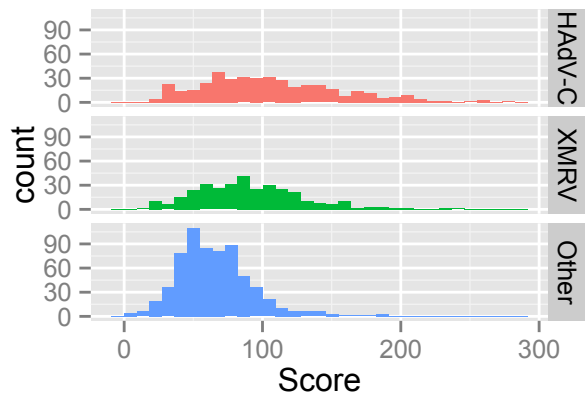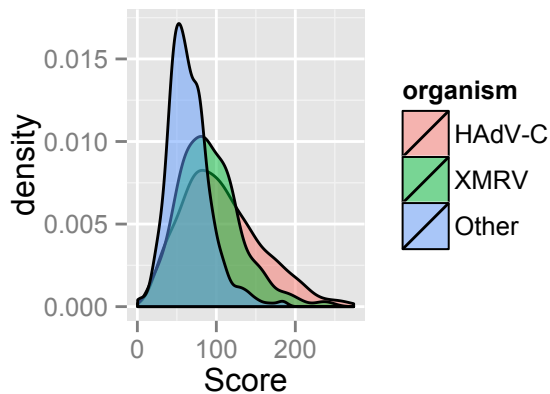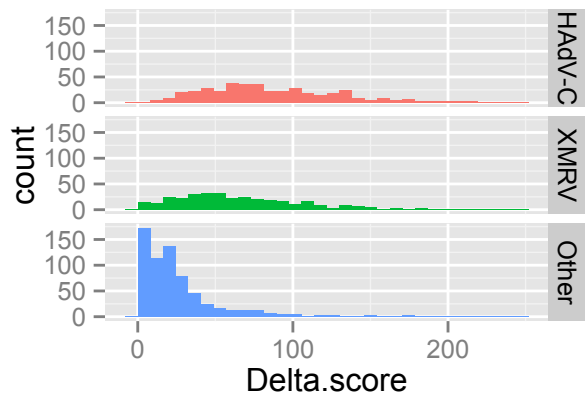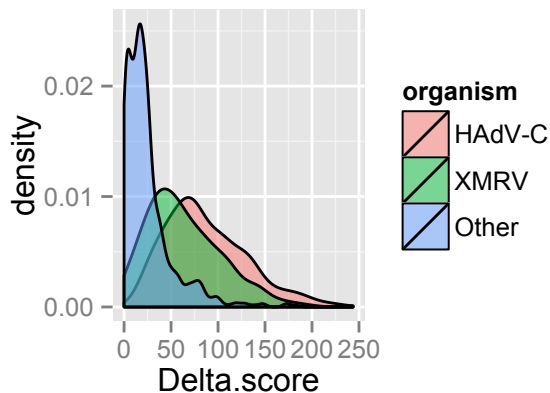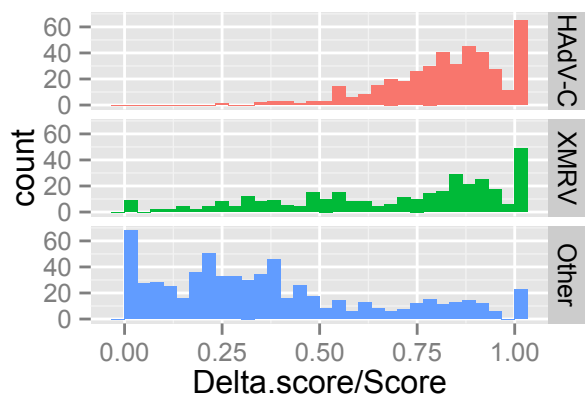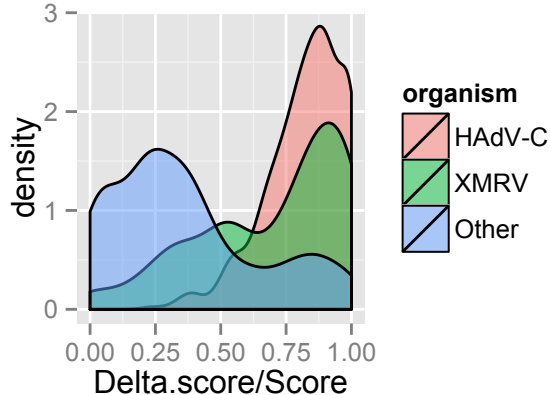

Supplement: Figure S1 — Histograms and density plots of Scores, Delta Scores and normalized Delta Scores (Delta Score divided by Score) for viral peptides identified in proteomes of eleven cell lines by Andromeda search engine at 1% FDR threshold by matching MS/MS spectra against concatenated human-viral sequence database. Abbreviations: HAdvC – human adenovirus C identified in HEK293 cell line, XMRV – xenotropic murine leukemia virus-related virus identified in LNCaP cell line, Other – other peptide-spectral matches from eleven cell lines uniquely attributed to viral peptides. (PDF) [file pone.0091433.s001.pdf]
